# The protective role of autophagy in experimental osteoarthritis, and the therapeutic effects of Torin 1 on osteoarthritis by activating autophagy

**DOI:** 10.1186/s12891-016-0995-x

**Published:** 2016-04-06

**Authors:** Ni-Tao Cheng, Ai Guo, Hai Meng

**Affiliations:** Beijing Friendship Hospital, Capital Medical University, Beijing, China

**Keywords:** Autophagy, Osteoarthritis, Cartilage degeneration, Torin 1, Protective role

## Abstract

**Background:**

Recent studies have shown that autophagy was associated with the development of osteoarthritis (OA), the purpose of this research was to determine the exact role of autophagy in OA and investigate effective therapeutic drugs to inhibit the pathological progression of OA.

**Methods:**

In this study, a cellular OA model was generated by stimulating SW1353 cells with IL-1β and a rabbit OA model was established by intra-articular injection of collagenase, followed by treatment with Torin 1 or 3-Methyladenine (3-MA). The mRNA expression levels of VEGF, MMP-13 and TIMP-1 were determined by quantitative real-time PCR. The caitilage degeneration was examined by histological evaluation, chondrocytes degeneration and autophagosomes were observed by transmission electron microscopy. Expression levels of Beclin-1 and LC3 were evaluated by western blotting and immunofluorescence.

**Results:**

The degeneration of SW 1353 cells, cartilage and chondrocytes was related to the loss of autophagy in experimental OA. 3-MA increased the severity of degeneration of cells and cartilage by autophagy inhibition, while Torin 1 reduced that by autophagy activation.

**Conclusions:**

The loss of autophagy is linked with the experimental OA and autophagy may play a protective role in the pathogenesis of OA. Treatment of Torin 1 can inhibit the degenerative changes of experimental OA by activating autophagy and it may be a useful therapeutic drug for OA.

**Electronic supplementary material:**

The online version of this article (doi:10.1186/s12891-016-0995-x) contains supplementary material, which is available to authorized users.

## Background

Autophagy is a cellular self-protection mechanism [1, 2] by removing damaged organelles and intracellular unfolded proteins [3]. It has been suggested to remain cellular homeostasis under stress conditions including xenobiotics, oxidants, infection and hypoxia [4]. However, autophagy has dual role in cell mechanism and homeostasis with both beneficial and pathogenic effects [5]. It may protect against numerous diseases including neurodegeneration, myopathy, liver disease and diabetes [6], while excessive autophagy can cause autophagic cell death [7, 8], and recent reports have shown that suppression of autophagy may lead to tissue degeneration [9, 10].

There are three major subtypes of autophagy: macroautophagy (normally called autophagy) [11], microautophagy [12] and chaperone-mediated autophagy [13]. Mitophagy plays an important role in the clearance of dysfunctioned mitochondria [14]. Autophagy mentioned in this paper is macroautophagy.

The most important characteristic of autophagy is the formation of autophagosomes [15], they fuse with lysosomes to form autolysosomes, and then the contents in autolysosomes will be degraded by lysosomal enzyme [16]. In this process, the expression levels of Beclin-1 and the LC3-II/LC3-I ratio are increased [17, 18]. Beclin-1 interacts with a multi-protein complex containing Vps34, a class III phosphatidylinositol-3 kinase (PI3KC3) [19] to initiate the formation of autophagosome. LC3 plays an important role in the autophagosome elongation and maturation [20].

Osteoarthritis (OA), also known as hypertrophic arthritis, is a degenerative joint disease and has become a prevalent clinical disorder in the elder [21]. Research has shown that many growth factors, cytokines and enzymes such as Interleukin (IL)-1β [22], metalloproteinases (MMPs) [23], inhibitors of metalloproteinase (TIMP)-1 [24] and collagenase [25] are closely related to the pathogenesis of OA. In addition, angiogenesis has been suggested to play important role in degeneration of articular cartilage after OA, and the level of vascular endothelial growth factor (VEGF) in OA cartilage is increased [26]. Because of these findings, IL-1β and collagenase are often applied to produce OA model for in-vitro and in-vivo studies [27, 28], while VEGF, MMPs and TIMP-1 are used to detect the degree of chondrocytes degeneration.

OA is mainly characterized by the death of chondrocytes and cartilage degeneration [29]. Accumulating evidences suggest that autophagy is closely related to the pathogenesis of OA. Some studies have shown that autophagy may keep chondrocytes survival in articular cartilage, and the expression levels of Beclin-1 and LC3-II in knee OA patients or models are increased [30, 31], while results obtained by some other studies are completely opposite [32, 33]. Meanwhile, there are currently no available effective drugs and measures for the prevention and treatment for OA in the clinical.

In this study, a cellular OA model was generated by stimulating SW1353 cells with IL-1β and a rabbit OA model was established by intra-articular injection of collagenase, followed by treatment with Torin 1 (a chemical autophagy inducer) or 3-Methyladenine (3-MA, a chemical autophagy inhibitor). The mRNA expression levels of VEGF, MMP-13 and TIMP-1 were determined by quantitative real-time PCR. The caitilage degeneration was examined by histological evaluation, chondrocytes degeneration and autophagosomes were observed by transmission electron microscopy. Expression levels of Beclin-1 and LC3 were evaluated by western blotting and immunofluorescence. The aim of this study was to determine the role of autophagy in OA and investigate the possible therapeutic drugs for OA.

## Methods

### Cellular OA model induced by IL-1β

Human chondrosarcoma SW 1353 cells were purchased from Institute of Life Science Cell Culture Center (Shanghai, China) and stimulated with IL-1β (10 ng/mL, Peprotech, US) to produce a cellular OA model. After stimulation for 1 h, the cells were treated with Torin 1 (250 nM, Selleck, US) or 3-MA (5 mM, Sigma, US) and incubated at 37 °C for 48 h. The cells without any treatment were used as controls.

### Quantitative real-time polymerase chain reaction (qRT-PCR)

Total RNA was extracted using the Trizol reagent (Invitrogen, US). The primers for VEGF, MMP-13, TIMP-1 and β-actin were: VEGF: forward, 5′-CCCACGTCAGAGAGCAACA-3′, reverse, 5′-TCACATCTGCTGTGCTGTAGG-3′; MMP-13: forward, 5′-CGACTTCTACCCATTTGA-3′, reverse, 5′-TAGCCTTTGGAACTACTTGTC-3′; TIMP-1: forward, 5′-AGATAGCCTGAATCCTGCC-3′, reverse, 5′-CTGGGTGGTAACTCTTTATTTC-3′; β-actin, forward, 5′-TCGACAACGGCTCCGGCAT-3′, reverse, 5′-AAGGTGTGGTGCCAGATTTTC-3′. All genes were analyzed using a 7500 Real Time PCR System (ABI, USA). Each sample was analyzed for 3 times and the mean values were calculated [34], and the test was repeated 3 times. The expression of target genes, relative to β-actin, was calculated using the 2^-ΔΔCT^ method.

### Immunofluorescence

Cells were seeded on a glass coverslip into a 6-well plate. After treatment, a DAPI immunofluorescence staining protocol was carried out. The rabbit anti-LC3 primary antibody was obtained from Cell Signaling Technology, the donkey anti-rabbit secondary antibody was obtained from Alexa Fluor. Each sample had 3 replicate wells and the test was repeated 3 times. The slides were examined under a confocal microscopy (Olympus, Japan).

### Rabbit OA model

Twenty healthy male New Zealand white rabbits at 3 months old (weighing 2.4–2.6 kg) were provided by the Animal Center of Capital Medical University (Beijing, China). All animal experiments were approved by the Institutional Animal Care and Use Committee. Rabbits were randomly assigned to three experimental groups and one control group. All animals were sacrificed at the end of 8 weeks after the initiation of experiment. The bilateral knee joints of animals of the first experimental group received intra-articularly injection with 0.5 ml saline containing 1.0 mg collagenase (type II, Sigma, US) to establish a rabbit OA model, the second experimental group received injection of 1.0 mg collagenase combined with 250 nM Torin 1, and the third experimental group received injection of 1.0 mg collagenase combined with 5 mM 3-MA. The injection was performed twice at day 1 and day 4 as reported [28]. Rabbits of the control group received twice injections of 0.5 ml normal saline. Penicillin and streptomycin were used to prevent infection by gluteal muscle injection, and side effects such as weight loss, diarrhea, anemia, proteinuria and allergy were not observed in this study. Cartilage samples dissected out from the knee joints of 5 rabbits in each group, giving a total of 10 joints, were used for histological evaluation, transmission electron microscope observation and western blotting, avoiding inclusion of both joints from 1 rabbit in each test.

### Histological evaluation

The lateral femoral condyles of each group were separated and prepared (*n* = 10). After decalcification and dehydration, the samples were paraffin-embedded and cut into 5 μm microsections in the sagittal plane, then stained with hemotoxylin and eosin (H&E). Grading of staining was evaluated by blinded observers using scoring according to the Mankin scoring system (Table 1) [35].

**Table 1:** Mankin scoring system

Articular cartilage	
(1) Structure (7 grades) (2) Cell (4 grades) (3) Safranin-O staining (5 grades) (4) Tidemark (2 grades)	

### Transmission electron microscopy

Cartilage from medial femoral condyles was fixed with 2.5 % glutaraldehyde in PBS and rinsed with PBS. After decalcification, the samples were further fixed with 1 % OsO_4_, rinsed with PBS, dehydrated by a series of ethanol, and incubated in isoamyl acetate. Finally, the samples were embedded in gold-palladium, cut into ultrathin sections, and observed under a transmission electron microscopy (TEM, Hitachi, Japan).

### Western blotting

Cartilage from tibial plateau was separated and prepared, each cartilage from 3 out of 10 tibial plateaus was pooled as one sample (*n* = 3), and the total protein of cells and cartilage in each group was extracted. The protein concentrations were measured using a bicinchoninic acid reagent assay (Thermo, US). Equal amount of proteins (50 μg) were separated by Tris-glycine gels (Sigma, US) and transferred onto nitrocellulose membranes (Millipore, US). The membranes were blocked with 5 % nonfat milk and incubated with antibodies specific for Beclin-1, LC3 (Cell signaling Technology, US) and β-actin (Santa Cruz, US). Then the nitrocellulose sheet was incubated with a horse-radish peroxidase (HRP)-conjugated secondary antibody (Zhongshan Golden Bridge Biotechnology, Beijing, China) and subjected to signal development by using enhanced chemiluminescence (ECL) substrate (Thermo, US). Each sample was analyzed three times and the mean gray values of immunoblot band were calculated [34].

### Statistical analysis

Statistical analysis was performed with one-way ANOVA using SPSS statistical software 19.0 (Chicago, IL, USA). The data was expressed as the mean ± standard deviation (SD). A difference of *P* value less than 0.05 was considered to be statistically significant.

## Results

### VEGF, MMP-13 and TIMP-1 expression

**Fig. 1:**
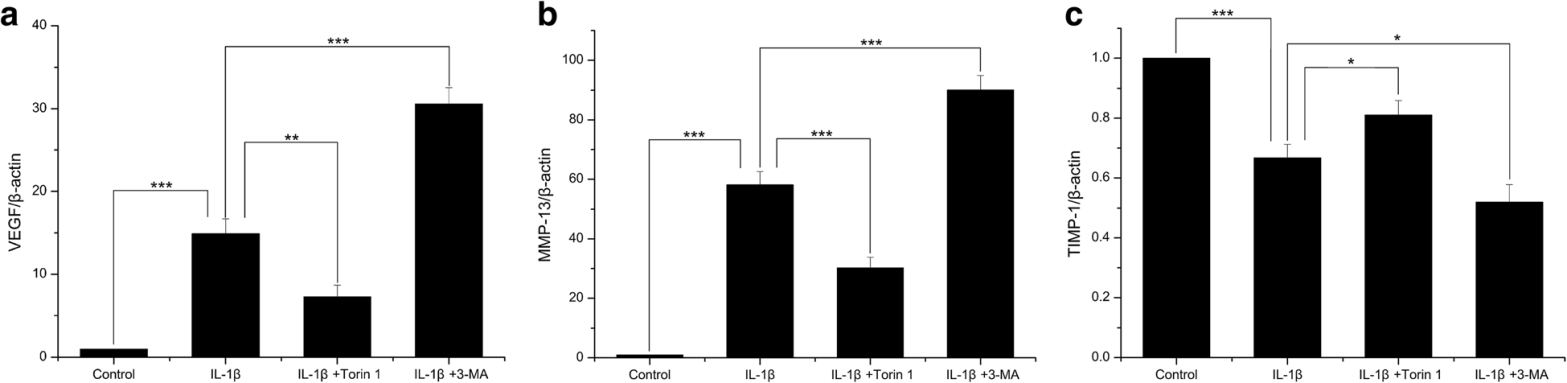
The mRNA expression of VEGF, MMP-13 and TIMP-1. **a**, **b** The mRNA expression levels of VEGF and MMP-13 in IL-1β-induced SW1353 cells were significantly increased compared with that in normal cells. **c** The mRNA expression level of TIMP-1 was significantly decreased. The increase in VEGF and MMP-13 expression, and decrease in TIMP-1 expression were significantly inhibited by Torin 1, while the effects of 3-MA were exactly opposite. *n* = 3 for each group, and the test was repeated 3 times. *P < 0.05, **P < 0.01, ***P < 0.001

The mRNA expression level of VEGF (Additional file 1) and MMP-13 (Aditional file 2) in IL-1β-induced SW1353 cells were significantly increased compared with that in normal cells (Both *P* < 0.001) (Fig. 1a and b), and the expression level of TIMP-1 (Additional file 3) was significantly decreased (*P* < 0.001) (Fig. 1c). In addition, the increase in VEGF and MMP-13 expression, and decrease in TIMP-1 expression were significantly inhibited by Torin 1 (*P* = 0.002, <0.001 and = 0.025), while the effects of 3-MA were exactly opposite (*P* < 0.001, <0.001 and = 0.023).

### Beclin-1 and LC3 expression in SW1353 cells

**Fig. 2:**
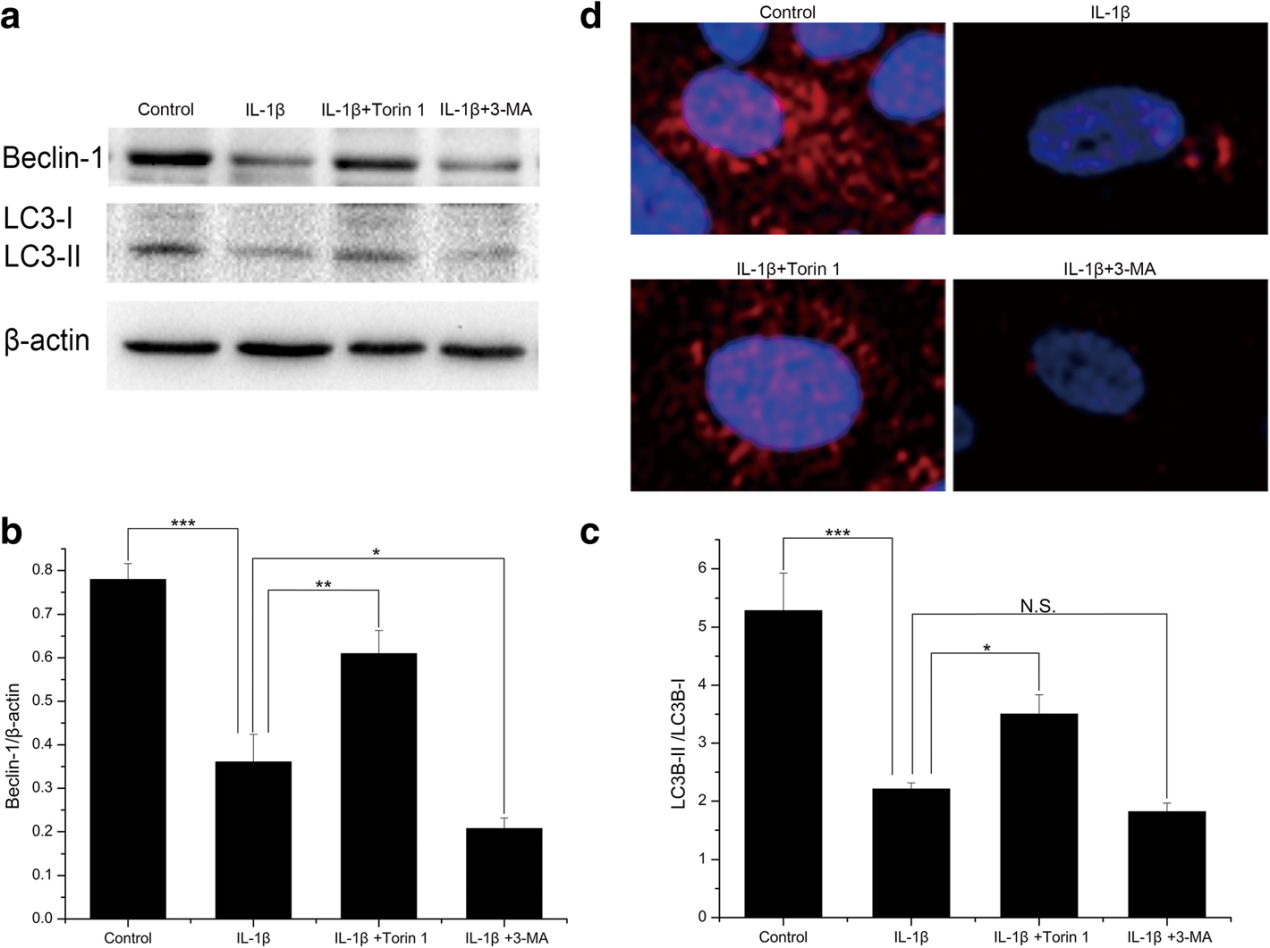
The expression of Beclin-1 and LC3 in SW1353 cells. **a** The protein expression of Beclin-1 and LC3 evaluated by western blotting. **b**, **c** Quantitative analysis showed that the protein expression of Beclin-1 and the LC3-II/LC3-I ratio were decreased in IL-1β-induced SW1353 cells compared with that in normal cells. Both the expression levels of Beclin-1 and the LC3-II/LC3-I ratio were much higher in cells treated with IL-1β in combination with Torin 1 than in cells only treated with IL-1β, while they were lower in cells treated with IL-1β in combination with 3-MA. *n* = 3 for each group. **d** The expression of LC3 evaluated by immunofluorescence. LC3 was expressed in normal cells and decreased in IL-1β treated SW1353 cells. Moreover, the expression of LC3 was higher in cells treated with IL-1β in combination with Torin 1 and lower in cells treated with IL-1β in combination with 3-MA. *n* = 3 for each group, and the test was repeated 3 times. *P < 0.05, **P < 0.01, ***P < 0.001

Based on above results, some autophagy-related proteins were detected (Fig. 2a). The protein expression of Beclin-1 (Additional file 4) and the LC3-II/LC3-I ratio (Additional file 5) were decreased in IL-1β-induced SW1353 cells compared with that in normal cells (Both *P* < 0.001). Both the expression levels of Beclin-1 and the LC3-II/LC3-I ratio were much higher in cells treated with IL-1β in combination with Torin 1 than in cells only treated with IL-1β (*P* = 0.001 and 0.019), while they were lower in cells treated with IL-1β in combination with 3-MA (*P* = 0.026 and 0.659) (Fig. 2b, c). We further evaluated LC3 protein expression by immunofluorescence and found that LC3 was expressed in normal cells and decreased in IL-1β treated SW1353 cells. Moreover, the expression of LC3 was higher in cells treated with IL-1β in combination with Torin 1, and lower in cells treated with IL-1β in combination with 3-MA than in cells only treated with IL-1β (Fig. 2d). The results obtained by immunofluorescence were consistent with western blotting.

### Histological changes

**Fig. 3:**
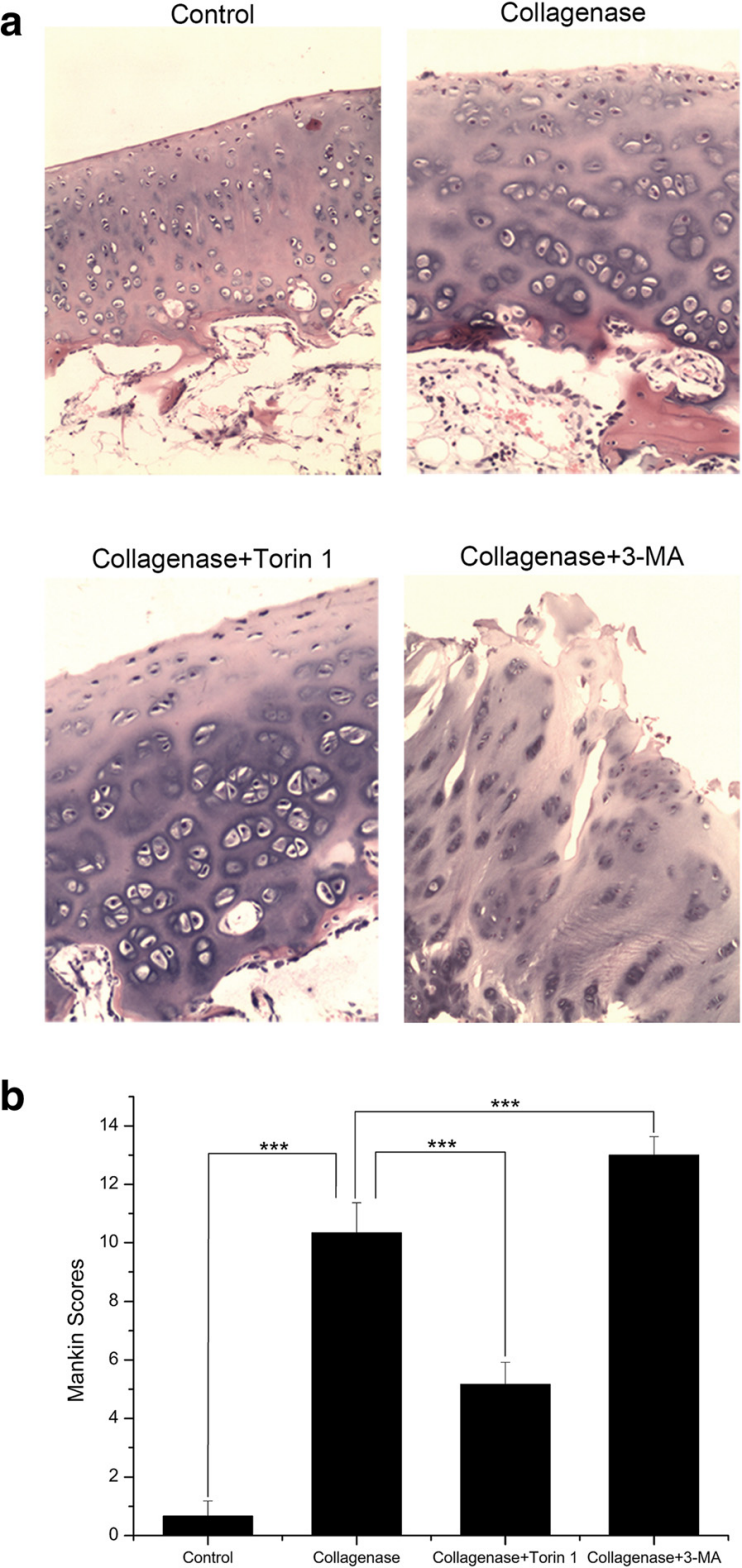
Histological changes in cartilage and Mankin scores. **a** In the control group, the cartilage was not degenerated and its surface was smooth. In the collagenase-injected group, the loss of cartilage had extended to the radial zone, cell cloning of chondrocytes and reduced stain ability were apparent in the radial zone. In the Torin 1-treated group, the loss of cartilage had only extended to the transitional zone, disappearance of cells in the transitional zone, cell cloning in the radial zone and reduced stain ability were observed. But in the 3-MA-treated group, much more clefts and loss of cartilage and seriously reduced stain ability were seen in the radial zone. **b** The scores of the collagenase-injected group were higher than those of the control group, and the scores were lower in the Torin 1-treated group but higher in the 3-MA-treated group when compared with collagenase-injected group. *n* = 10 for each group. ***P < 0.001 (Magnification, 100×)

In the control group, the cartilage was not degenerated and its surface was smooth. In the collagenase-injected group, the loss of cartilage had extended to the radial zone, cell cloning of chondrocytes and reduced stain ability were apparent in the radial zone. In the Torin 1-treated group, the loss of cartilage had only extended to the transitional zone, disappearance of cells in the transitional zone, cell cloning in the radial zone and reduced stain ability were observed. But in the 3-MA-treated group, much more clefts and loss of cartilage and seriously reduced stain ability were seen in the radial zone (Fig. 3a). The mankin scores (Additional file 6) of the collagenase-injected group were higher than those of the control group (*P* < 0.001), and the scores were lower in the Torin 1-treated group but higher in the 3-MA-treated group when compared with collagenase-injected group (Both *P* < 0.001) (Fig. 3b and Table 2).

**Table 2:** Mankin scores

Mankin scores	
Control Collagenase Collagenase + Torin 1 Collagenase + 3-MA	0.67 ± 0.52 10.33 ± 1.03 5.17 ± 0.75 13.00 ± 0.63

Each data represents the mean ± SD (*n* = 10)

### TEM observation

**Fig. 4:**
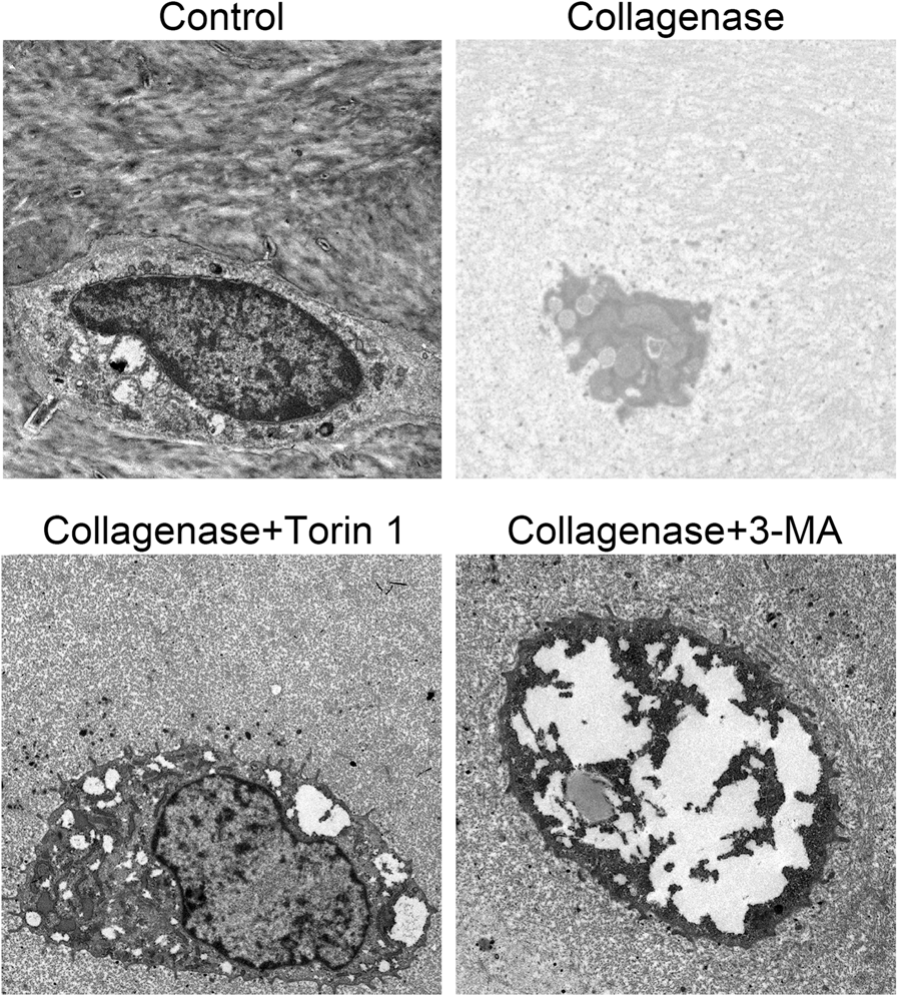
TEM observation and autophagosomes evaluation. In the control group, chondrocytes were located in the lacunae and contained round nuclei with discontinuous nuclear membranes, some vesicles were observed in the cytoplasm. In the collagenase-injected group, typical condensed chondrocytes with several autophagic vacuoles were observed, the number of autophagosomes in collagenase treated chondrocytes was less than the controls, and the nuclei of chondrocytes were disappeared. In the Torin 1-treated group, autophagy became more pronounced and much more autophagosomes were observed in chondrocytes. Meanwhile, abundant rough endoplasmic reticulum (RER) and a few other organelles were found in the cytoplasm. Moreover, the nuclei of chondrocytes were present, and condensed chromatin produced a convoluted pattern. In the 3-MA-treated group, the contents in chondrocytes had been substantially degraded. Disappeared nuclei, a number of cell debris and a large autophagosome were observed. (Bar, 1 μm)

Chondrocytes degeneration and autophagosomes were examined by TEM (Fig. 4). In the control group, chondrocytes were located in the lacunae and contained round nuclei with discontinuous nuclear membranes, some vesicles were observed in the cytoplasm. In the collagenase-injected group, typical condensed chondrocytes with several autophagic vacuoles were observed, the number of autophagosomes in collagenase treated chondrocytes was less than the controls, and the nuclei of chondrocytes were disappeared. In the Torin 1-treated group, autophagy became more pronounced and much more autophagosomes were observed in chondrocytes. Meanwhile, abundant rough endoplasmic reticulum (RER) and a few other organelles were found in the cytoplasm. Moreover, the nuclei of chondrocytes were present, and condensed chromatin produced a convoluted pattern. In the 3-MA-treated group, the contents in chondrocytes had been substantially degraded. Disappeared nuclei, a number of cell debris and a large autophagosome were observed.

### Beclin-1 and LC3 expression in rabbit OA model

**Fig. 5:**
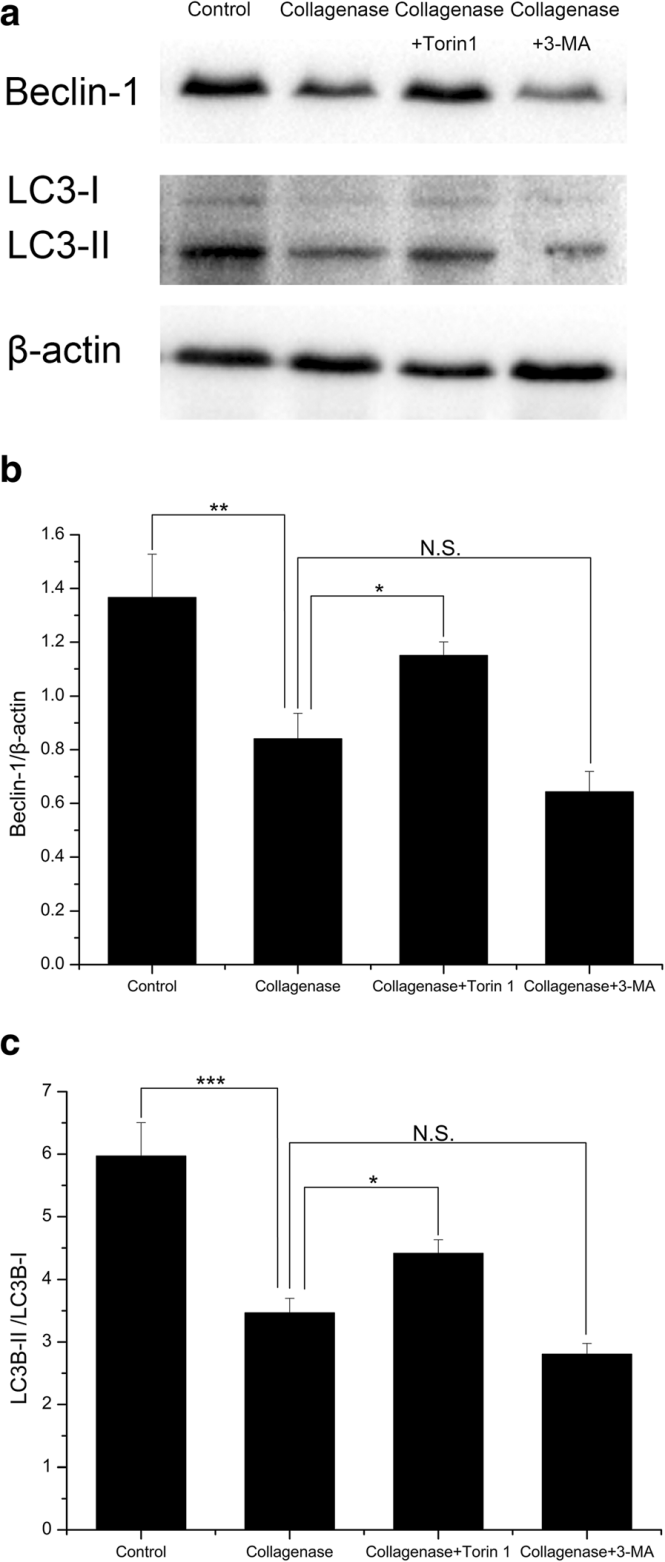
The Beclin-1 and LC3 expression in rabbit OA model. **a** The protein expression of Beclin-1 and LC3 evaluated by western blotting. **b**, **c** Quantitative analysis showed a significantly reduction of Beclin-1 expression and the LC3B-II/LC3B-I ratio in the collagenase-injected group compared with the control group. Similarly, expression levels of Beclin-1 and the LC3B-II/LC3B-I ratio were much higher in the Torin 1-treated group than in the collagenase-injected group, while they were lower in the 3-MA-treated group. *n* = 3 for each group. *P < 0.05, **P < 0.01, ***P < 0.001

Western blotting analysis showed a significantly reduction of Beclin-1 expression (Additional file 7) and the LC3-II/LC3-I ratio (Additional file 8) in the collagenase-injected group compared with the control group (*P* = 0.002 and <0.001). Similarly, expression levels of Beclin-1 and the LC3-II/LC3-I ratio were much higher in the Torin 1-treated group than in the collagenase-injected group (*P* = 0.041 and 0.044), while they were lower in the 3-MA-treated group (*P* = 0.225 and 0.178) (Fig. 5a, b and c).

## Discussion

OA has become one of the most common chronic joint disease in clinical [36], the mechanism and effective treatment to inhibit the pathological progression remain to be the hot issues in the field of medical research. For these reasons, many kinds of OA models have been produced using different materials and methods, such as Hulth model [37], joint fixation [38] and intra-articular injection of drugs [39, 40]. These models are thought to be useful for the investigation of OA pathogenesis and treatment.

Reports have shown that IL-1β and collagenase are involved in the pathogenesis of OA [41, 42]. Based on these studies, IL-1β is often used to build cellular OA model [43, 44], and collagenase is used to establish OA models by intra-articular injection [27, 28, 45]. In our previous reports, we stimulated SW1353 cells with IL-1β, and successfully induced degenerative changes in SW1353 cells, such as loss of cell viability, increased synthesis of MMPs, we determined that IL-1β could promote the occurrence of OA by activating the mitogen-activated protein kinase (MAPK) pathway [46]. In the present study, we still produce a cellular OA model by IL-1β, and establish a rabbit OA model by intra-articular injection of collagenase. We notice the significantly increased expression levels of VEGF and MMP-13, and the significantly decreased expression level of TIMP-1 in SW1353 cells, accompanied by the degeneration of cartilage and chondrocytes in knee joints. These observations demonstrate degenerative changes in SW1353 cells and knee joints separately induced by IL-1β and collagenase, but the physiologic differences between human and model could still make differences between the results with the actual situation.

Autophagy plays an essential role in cellular metabolism and homeostasis and can keep cell survival under stress conditions [47]. In addition, autophagy has also been considered to take part in numerous diseases, including a variety of orthopedic disorders, such as degradation of Meckel's cartilage [48], intervertebral disc degeneration [49] and OA [50, 51]. But recent results about the relationship between autophagy and OA are sometimes contradictory [30–33]. In this study, we detect the expression levels of autophagy-related proteins, Beclin-1 and LC3, and we determine the expression levels of them are significantly reduced both in IL-1β-induced cells and collagenase-induced knee joints. Meanwhile, TEM observation show typical condensed chondrocytes with several autophagic vacuoles, and the collagenase treated chondrocytes have less autophagosomes than the controls. These results demonstrate that the loss of autophagy is linked with the experimental OA.

To determine the exact role of autophagy in OA and investigate effective therapeutic drugs to inhibit the pathological progression of OA, we add the treatment of Torin 1 and 3-MA in celluar OA model and rabbit OA model. We notice that the expression levels of Beclin-1 and LC3 are enhanced by Torin 1, the autophagy become more pronounced and much more autophagosomes are observed in chondrocytes of cartilage, and the degenerative changes in cells and knee joints are inhibited, while treatment of 3-MA leads to completely opposite results. Thus the present observations indicate that autophagy may play a protective role in the pathogenesis of OA. What’s more, treatment of Torin 1 can inhibit the degenerative changes of experimental OA by activating autophagy and it may be a useful therapeutic drug for OA.

## Conclusions

In summary, our results demonstrated that the degeneration of SW 1353 cells, cartilage and chondrocytes was related to the loss of autophagy in experimental OA, and 3-MA increased the severity of degeneration of cells and cartilage by autophagy inhibition, while Torin 1 reduced that by autophagy activation. These observations indicate that the loss of autophagy is linked with the experimental OA and autophagy may play a protective role in the pathogenesis of OA. Furthermore, Treatment of Torin 1 can inhibit the degenerative changes of experimental OA by activating autophagy and it may be a useful therapeutic drug for OA.

### Availability of data and materials

The datasets supporting the conclusions of this article are included within the article and its additional files.

## Additional files

Additional file 1: VEGFR2. (XLSX 10 kb) Additional file 2: MMP-13R2. (XLSX 10 kb) Additional file 3: TIMP-1R2. (XLSX 10 kb) Additional file 4: Beclin-1 in SW1353 cellsR2. (XLSX 10 kb) Additional file 5: LC3 in SW1353 cellsR2. (XLSX 10 kb) Additional file 6: The Mankin ScoresR2. (XLSX 10 kb) Additional file 7: Beclin-1 in rabbit OA modelR2. (XLSX 10 kb) Additional file 8: LC3 in rabbit OA modelR2. (XLSX 10 kb)

## Abbreviations

3-MA: 3-Methyladenine
ECL: enhanced chemiluminescence
H&E: hemotoxylin and eosin
HRP: horse-radish peroxidase
IL-1β: Interleukin-1β
LC3: light chain 3
MAPK: mitogen-activated protein kinase
MMP-13: matrix metallopeptidase-13
OA: osteoarthritis
PI3KC3: class III phosphatidylinositol-3 kinase
qRT-PCR: Quantitative real-time polymerase chain reaction
RER: rough endoplasmic reticulum
SD: standard deviation
TEM: transmission electron microscopy
TIMP-1: inhibitors of metalloproteinase-1
VEGF: vascular endothelial growth factor
